# Medical Education and Health Care, Is There a Conflict?

**Published:** 1988-02

**Authors:** Charles Clarke

**Affiliations:** Solicitor of the Supreme Court late Chairman of Bristol and Weston Health District


### Medical Education & Health Care—is there a conflict?

**Charles Clarke CBE, D. Litt.**
Solicitor of the Supreme Court
late Chairman of Bristol and Weston Health District

**EDWARD LONG FOX MEMORIAL LECTURE, 1986**

It might be thought that the best of Medical Education and the best of Health Care were synonymous, and that what was best for one was best for the other, but it has not always been so, and some may doubt whether it ever will be.

The tensions which arise between Medical School and Teaching Hospital staff cannot be dismissed lightly and another area, that of the nature and composition of Medical Education deserves fuller consideration.

The difficulties which arise between clinical medical staff employed in the National Health Service and the academic staff, generally of equivalent Consultant rank, who are actually employed through Medical Schools by the Universities, on whose staff they are, are complex. In the last thirteen years, there has been a gradual decline in funds, both from the Exchequer to the N.H.S. and from the University Grants Committee to the Medical Schools. Everybody here is unhappily familiar with the results. One of the problems is that nobody is quite sure where the goal posts are, and sometimes one wonders whether this state of affairs is impishly deliberate. Section 12(3) of the N.H.S. Act 1946 says that it is the duty of the Board of Governors of every Teaching Hospital "to provide, for the University with which that hospital is associated, such facilities as *appear* to the Minister to be required for clinical teaching and research". Apart from this delphic utterance and one or two sections which relate specifically to London University the Act is otherwise silent on Medical Education and very little of what appeared to the Minister appears to have been passed on. From the University side, we have almost less guidance. It comforts some people, but not all, to know that academic clinicians have to do the equivalent of six sessions a week of clinical work, in order to qualify for NHS distinction awards.

The academic year has advantages and disadvantages. There is no such thing, on the other hand, as the NHS year: it never ends.

On the vexed subject of comparability of rewards, it is hard to be dispassionate. I am acutely aware of the tensions existing in Medical Schools now on this subject, and of the consequent sheeps' eyes being cast at the right to browse in the lucrative fields of private practice. These feelings will have consequences if not alleviated, if indeed such consequences are not already in train.

The loss will fall, first, on the field of Research, and, later, on undergraduate Medical Schools, whose staff will become difficult to recruit. Excellent men and women have already gone over to the N.H.S. The views of some Politicians of all parties, and of some Senior Civil Servants, should be understood. They believe that the human body wears out in a little over the psalmists' three score years and ten, that only in cancer and heart disease does much remain to be done to allow most of us this expectation, and that scarce resources available for research are truly better employed in other fields: perhaps, Vice Chancellor, in those not long since under your able encouragement.

In that way, the argument runs, our economic prosperity, from which health care must of necessity be financed, can be made more sure.

In this conflict, which is not strictly within my brief tonight, it is impossible not to have a view after all these years, and for what it is worth, I will give it. It is the quality of life, and not the length of it, which matters. My vote should go for research into psychiatry and orthopaedics, unglamorous subjects compared with plastic hearts, but what a joy they can restore to many people's lives.

In my experience there has been a very great deal of valuable and fruitful co-operation in teaching and in research. Although it is written in no Consultant's contract that they have a duty to teach, they have willingly and cheerfully done so with great success. Academic staff have, in countless ways, contributed to the welfare and wellbeing of the hospitals and their patients. For example, they take their turn in on-call rotas, although there has been no legal requirement upon them to do so. I also pay glowing tribute to the share of the management and administrative responsibilities which academic staff have so freely and cheerfully and usefully given over many years.

This curious marriage, like all marriages, requires to be worked upon, and when it is, it is a splendid thing. That it works in Bristol and elsewhere is abundantly clear, and if, in the last year or two, the machinery has creaked slightly, everyone has reached for the oil until it is running smoothly again. Restraint and forebearance enable all of us to live with this, and see it thrive.

The fourth area of conflict and the one with which I propose to deal in greater detail has become, for me, the most interesting of all. On the one hand are the Medical Schools which are educating and training medical students, and turning out men and women qualified for the register, and fit to take their place to practice the honourable calling of medicine, and on the other hand, waiting to receive them, stands what may broadly be called Society, with an expectation of the doctors they are likely to meet, which may, or may not, be quite the same as those of the Medical Schools.

I am a great admirer and supporter of the Medical Profession. They are gifted, learned, compassionate people of great sensitivity, usually gentle and almost invariably kind. I am considering whether their training helps them in their future careers, to match the public expectation of them, and it seems likely that in one or two respects, it does not. I fear that the pressures upon the architects of their curricula oblige them to confine their formal studies to the presentation of facts, with some scientific principles as to consideration and evaluation of them, and that any specific guidance on matters other than technical has for many years been confined to emulation and precept. Many of my friends and acquaintances were asked what they expect of their doctors. All said competence, of course. But most of them put understanding very high, the application and reference of particular advice or treatment or referral to their own circumstances, and those of their families. While many spoke highly of General Practitioners and Consultants, a considerable number did not. They spoke of indifference, of secretiveness, of insensitivity in bereavement. I know that my family and I have been exceedingly fortunate in our experiences but perhaps there is a lesson for us all in that.

It is a privilege to speak in memory of so great a man as Edward Long Fox, who, living in a far from enlightened time, had not only an outstanding sympathy with his patients and their families, but also had the foresight to recognise that mental illness was not permanent, had the courage to do something about it, and the determination and staying power to succeed in that most laudable object. I am also deeply conscious of the honour to be the invitee of the Medico-Chirurgical Society, whose work over so many years, in the hands of successive dedicated men and women, has made so great a contribution to our Commonwealth.

Perhaps this is an occasion when I may say what I truly think.

It was a little over thirty years ago, in the mid-fifties, when Lord Moran made his unfortunate remark in relation to General Practitioners about the rungs of the ladder, and even Lord Platt in his report on the welfare of children in hospitals, as recently as 1958, felt it necessary to say that "we are unanimous in our opinion that the emotional needs of a child in hospital require constant consideration", and he went on to recommend the reorganisation of training of Medical students and nurses, so that they should concentrate, not only on the physical aspects of illness, but also on the child's emotional and mental needs, "of which, at the present time, far too little account is taken".

In the comments that many of my long-suffering medical and dental friends offered me, there has been a clear pattern. All, looking back on their student days, felt crammed with facts, too crammed. One had forgotten all his biochemistry, and most of his anatomy and physiology. He had never needed them. He had obtained, and held a Chair with distinction none the less. Too much is pushed into the poor students, said another, and there were a lot of comments on this theme.

But, I also found much to be glad of. Good standards are much more uniform now, one said. Notable all-round training, said another. Younger doctors are much more impressive, said a third, and their knowledge is more broadly based.

From one much experienced in the choice of general practitioners, came further reassurance. The foundation and development of the R.C.G.P. had been most beneficial, not only, he believed, on general medicine but on medicine generally. Vocational Training was now established, much to our good. They also now look for knowledge of practice management, business knowledge and awareness of staff relation, and expect to find them. He was persuaded that the younger doctors show more insight than was evident ten or fifteen years ago.

I turned, at the end of my investigation, to a close friend of mine who sits on the Education Committee of the G.M.C. and my hopes suffered a slight setback. He is a most reasonable man, with a deserved record of careful good in the public service. He felt that not only were the G.M.C.'s powers of regulation limited, but the criteria they used were sometimes inappropriate. The visiting Inspectors, almost invariably medically qualified, consistently reported that the taking of notes of case histories was poor. The humanities and behavioural sciences were poorly taught. Some Schools of Psychiatry were insensitive. Often, particularly in the London Schools, the Education Committee found little respect for the privacy and dignity of patients in any department. It seems to me, on carefully examining my notes of this conversation, that his is a counsel of perfection. Indeed it ought to be, for if it is to justify its existence, that is the standard for which the G.M.C. must strive. He agreed that the range of medical students' knowledge was vast, and that they can find usually things wrong without much difficulty. In these divergent currents, where is the eternal truth the student seeks? He wishes to become a good healer. Who warns him of the insidious pressures of the media, to which, nowadays, he is sure sooner or later to be exposed? How is he to deal with them? Who helps him to get the threat of legal liability for negligence into proper perspective? How does he see, and weigh up for himself, the true motives of one of the distinguished officers of one of the Royal Colleges, who can be just as devious or obtuse as the rest of us? What is the place of Health Education? When is it best simply to alleviate suffering, and not go all out for a cure?

Detachment, said a most distinguished Emeritus Professor to me sadly, is so difficult to reach. That is true in Life, not only in medicine, and it is equally difficult to learn.

I bring you no elixir tonight.

The legal profession, to which I am honoured to belong, is no better in training its students, or equipping them to meet the demands which, all too soon, will be thrust upon them. There are, all the same, some things which might, in all humility, be said.

How many times, in the last twenty years, looking at the C.V. of a senior registrar aspiring to become a Consultant, have I seen, under the heading outside interests, only hill-walking, squash, photography and DIY—indeed, many people do not even mention outside interests at all.

I take issue with Doctor Clements when he said that one should be careless of other objects. Zealots fill me with unease, for they can do much harm and achieve much distortion. Zealots and other obsessives have their place: I suppose Wilberforce was one. But so was Napoleon, and so was Adolf Hitler. How good it is to see, amongst other interests, English Literature, or Drama, or Art, in any of its splendid forms, or Travel, or Gardening, or Wine! Could influential men allow time for these, and encourage their pursuit? I was delighted to read one article in a learned journal by a distinguished Emeritus Professor here, whom it is sorrow to me not to mention by name. He there exposed, and compared, some of the heroes of old classic literature to the medical knowledge of today. It was profound, it was a feast.

Leadership is best acquired the hard way, in practice. Difficult persuasion is a great former of character. Discussion and debate have, in my view, a most helpful effect of broadening the mind. Do medical students have time for this? Could time, perhaps, be found?

The character wilts or flowers in adversity, and a little adversity is no bad thing. But character flowers also in fellowship, and joy.

Fellowship there is aplenty in our medical school, I do not doubt, but how much chance do we get to encourage innocent, plain joy?

And conversation. How much can be learned, to our infinite delight, in listening to, and perhaps taking part in, stimulating conversation. Then combine it with a modicum of good wine, and what fun can be had. But the conversation must be broadly-based. One of our older Emeritus Professors told me that when he took a houseman's job in Liverpool in the twenties, he was told in which Street (but not which house) to seek lodgings, and advised to confine his social intercourse to other medical men. A great many ordinary people, in many walks of life, are the better because they never lived by that precept. Many doctors are only too well aware of the value of this broad outlook, and have taken active and most praise-worthy steps to communicate with other professions and in wider fields. For example, the various Medical Centres are admirable, and the work they do, most constructive. Other examples are to be found in the various Medico-Legal Societies, and there is a great deal more of that sort.

I am truly grateful to the many people who have helped me to prepare what I have said to you tonight, and most appreciative of the honour of being invited to say it.

Let Hippocrates, who, I believe, still has something to offer, have the last word: "Between wisdom and medicine there is no gulf fixed; in fact medicine possesses all the qualities that make for wisdom. For a Physician who is a lover of wisdom is the equal of a God".

